# Complete Genome Sequence of the Industrial Bacterium *Ketogulonicigenium vulgare* SKV

**DOI:** 10.1128/genomeA.01426-16

**Published:** 2016-12-22

**Authors:** Nan Jia, Ming-Zhu Ding, Yu-Zhang Du, Shuai Feng, Feng Gao, Ying-Jin Yuan

**Affiliations:** a Key Laboratory of Systems Bioengineering (Ministry of Education), School of Chemical Engineering and Technology, Tianjin University, Tianjin, People’s Republic of China; b SynBio Research Platform, Collaborative Innovation Centre of Chemical Science and Engineering (Tianjin), School of Chemical Engineering and Technology, Tianjin University, Tianjin, People’s Republic of China; c Shandong Luwei Pharmaceutical Co., Ltd., Zibo, Shandong, People’s Republic of China; d Department of Physics, Tianjin University, Tianjin, People’s Republic of China

## Abstract

*Ketogulonicigenium vulgare* has been widely used in vitamin C two-step fermentation, which converts l-sorbose to 2-keto-l-gluonic acid. Here, the complete genome of *K. vulgare* SKV, which performs better fermentation production than *K. vulgare* Hbe602, is deciphered to understand the key differences in metabolism between *K. vulgare* strains SKV and Hbe602.

## GENOME ANNOUNCEMENT

*Ketogulonicigenium vulgare* was identified as a member of the *Proteobacteria* (1). It can convert l-sorbose to 2-keto-l-gulonic acid (2-KGA), the precursor of vitamin C (2). Mono-cultured* K. vulgare* grows poorly and the *Bacillus* spp. are usually cocultivated with it to achieve a high 2-KGA yield (3). Previously, we published the genome of *K. vulgare* Hbe602 (4). Our research showed that combinational expression of sorbose/sorbosone dehydrogenases and cofactor pyrroloquinoline quinone in *K. vulgare* Hbe602 could enhance 2-KGA production properly (5). It was interesting to remark that industrial *K. vulgare* performed better 2-KGA production than* K. vulgare* Hbe602 when they were cocultured with *Bacillus thuringiensis* Bc601 (6).

*K. vulgare* strain SKV (Shandong Luwei Pharmaceutical Co., Ltd.) was cultured in 250-ml flasks at 30°C and 250 rpm for 35 h. The seed medium contains 3 g/L beef extract, 3 g/L yeast powder, 3 g/L corn steep liquor, 0.2 g/L MgSO_4_, 1 g/L KH_2_PO_4_, 1 g/L urea, and 10 g/L peptone. The genomic DNA was isolated using the SDS method. The genome of *K. vulgare* SKV was sequenced by single-molecule real-time (SMRT) technology (Beijing Novogene Bioinformatics Technology Co., Ltd.). SMRT Analysis version 2.3.0 was used to filter low-quality reads, and the filtered reads were assembled to generate one contig without gaps.

The genome of *K. vulgare* SKV was annotated through the NCBI Prokaryotic Genome Annotation Pipeline (7) and using BLAST (8) against the Kyoto Encyclopedia of Genes and Genomes (KEGG) database (9) and the Clusters of Orthologous Groups (COG) of proteins database (10). The tRNAs and rRNAs were predicted by tRNAscan (11) and RNAmmer (12), respectively. The origin of replication (*oriC*) and putative DnaA boxes were identified using Ori-Finder (13). GC-Profile was used to identify the GC content variation in DNA sequences (14).

The genome of *K. vulgare* SKV consists of one circular chromosome (2,764,573 bp) and one circular plasmid (267,949 bp). The sequence difference between *K. vulgare* SKV and Hbe602 was analyzed using BLAST, and the sequence similarity between the chromosomes of* K. vulgare* SKV and Hbe602 is more than 99%. Additionally, the plasmid in *K. vulgare* SKV is almost the same as plasmid 1 in Hbe602. The better ability of 2-KGA production in *K. vulgare* SKV may be due to the loss of plasmid 2 in Hbe602. Plasmid 2 of *K. vulgare* Hbe602 encodes 211 proteins, which are mainly related to transport systems, transcriptional regulators, and dehydrogenases. We identified that overexpression of l-sorbosone dehydrogenase (GI: 939479492) in plasmid 2 of *K. vulgare* Hbe602 produced an obvious byproduct in *K. vulgare* (15). Compared with *K. vulgare* Hbe602, the loss of dehydrogenases may lead to the higher 2-KGA production. We hope these findings can provide insight into the metabolism and gene targets for the strain improvement of *K. vulgare*.

### Accession number(s).

The sequence of the *K. vulgare* SKV genome has been deposited at DDBJ/EMBL/GenBank under the GenBank accession numbers CP016592 (chromosome) and CP016593 (plasmid).
